# On the Response of Ultralean Combustion of CH_4_/H_2_ Blends in a Porous Burner to Fluctuations in Fuel Flow—an Experimental Investigation

**DOI:** 10.1021/acs.energyfuels.1c00081

**Published:** 2021-05-04

**Authors:** Rabeeah Habib, Bijan Yadollahi, Ali Saeed, Mohammad Hossein Doranehgard, Nader Karimi

**Affiliations:** † James Watt School of Engineering, University of Glasgow, Glasgow G12 8QQ, U.K.; ‡ Department of Civil and Environmental Engineering, School of Mining and Petroleum Engineering, University of Alberta, Edmonton, Alberta T6G 1H9, Canada; § School of Engineering and Materials Science, Queen Mary University of London, London E1 4NS, U.K.

## Abstract

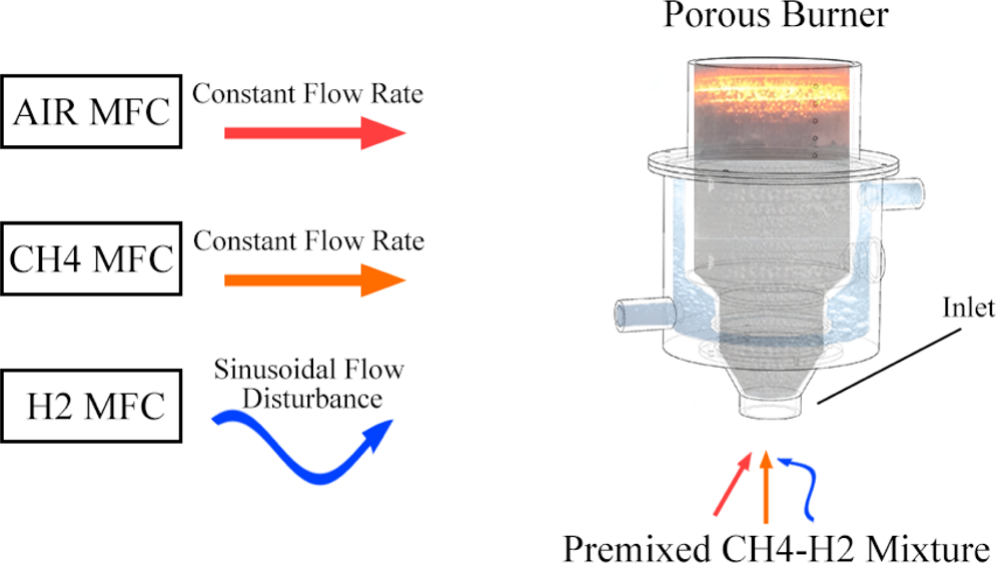

Fluctuations in the fuel flow rate may occur in practical combustion systems and result in flame destabilization. This is particularly problematic in lean and ultralean modes of burner operation. In this study, the response of a ceramic porous burner to fluctuations in the flow rate of different blends of methane and hydrogen is investigated experimentally. Prior to injection into the porous burner, the fuel blend is premixed with air at equivalence ratios below 0.275. The fuel streams are measured and controlled separately by programmable mass flow controllers, which impose sinusoidal fluctuations on the flow rates. To replicate realistic fluctuations in the fuel flow rate, the period of oscillations is chosen to be on the order of minutes. The temperature inside the ceramic foam is measured using five thermocouples located at the center of the working section of the burner. The flame embedded in porous media is imaged while the fuel flow is modulated. Analysis of the flame pictures and temperature traces shows that the forced oscillation of the fuel mixture leads to flame movement within the burner. This movement is found to act in accordance with the fluctuations in methane and hydrogen flows for both CH_4_(90%)–H_2_(10%) and CH_4_(70%)–H_2_(30%) mixtures. However, both fuel mixtures are noted to be rather insensitive to hydrogen flow fluctuation with a modulation amplitude below 30% of the steady flow. For the CH_4_(70%)–H_2_(30%) mixture, the flame in the porous medium can be modulated by fluctuations between 0 and 30% of steady methane flow without any noticeable flame destabilization.

## 1 Introduction

Natural gas is currently used widely throughout the world and is expected to continue being a major source of energy in the foreseeable future.^1^ Production of carbon dioxide by combustion of natural gas is smaller than that of other fossil fuels. Nonetheless, this still poses a substantial concern, hence there exist active plans for decarbonization of gas grids.^2,3^ Injection of hydrogen to natural gas pipelines has been identified as a practical approach to reduce carbon emissions.^4,5^ As a result, in recent years, there has been a surge of research interest in the combustion of hydrogen and methane mixtures, for example, refs (6, 7). Although these studies^8,9^ have provided a wealth of insights into the problem, they are chiefly focused on lean premixed and partially premixed flames.^10^ This makes them pertinent to high-temperature applications typically more than 1500 K. There are, however, an increasing number of applications in which high temperatures are not needed, and generation of heat at moderate temperatures is preferred.^11^ Ultralean combustion may provide a solution to the problem of heat generation at moderate temperatures.^12,13^ Yet, some important challenges should be addressed first. These include management of carbon monoxide emissions and flame stability issues.^14,15^ The current work is focused on the latter through analysis of ultralean combustion of CH_4_–H_2_ blends in a porous burner.

An ultralean blend of air and fuel is inherently a low-calorific value mixture.^16^ The resultant reduction in the temperature makes the flame susceptible to blow-off and extinction.^17^ It has been already shown that porous burners can significantly enhance the flame stability.^18^ Excellent thermal properties of a porous ceramic foam enable combustion of premixed low calorific fuels that would not be otherwise possible.^19,20^ At present, the use of porous burners can be found in several engineering applications.^21,22^ These include propulsion and gas turbine systems,^23,24^ heat exchangers,^25^ and chemical processing.^26^ A fundamental issue in stability of combustion system operation under lean and ultralean conditions is their response to temporal changes in the fuel flow rate.^27^ Such changes can occur during the start-up and shut-down and can be also encountered when the fuel composition varies.^27,28^ Furthermore, due to the small flow rates of hydrogen and methane in ultralean burners, mechanical defects can cause flow fluctuations. Robustness of the ultralean burner is heavily influenced by its response to such fluctuations in the fuel flow rate.

The literature on combustion in chemically inert porous media is rather large (see e.g. refs (29, 30)), and reviewers of literature can be found in refs (31, 32). Importantly, however, most of the existing studies on combustion in porous media are concentrated on steady-state conditions.^33,34^ The majority of experiments in this area evaluated the burner performance,^35,36^ and only a few considered the ultralean conditions.^37,38^ Thus, a very small fraction of the vast literature on reacting flows in porous media is related to the unsteady combustion and flame stabilization issues. In the following, these studies are briefly discussed. Furthermore, due to the peculiarities of hydrogen flames, combustion of hydrogen in porous media is also included in the discussion.

Kakutkina et al.^39^ experimentally investigated hydrogen–air combustion inside a porous burner. The mixture was ignited upstream of a quartz tube containing a porous medium, which also provided partial optical access to the flame.^39^ The flame movement was recorded via a digital camera, and the temperature was measured by a thermocouple at the designated parameters of the hydrogen–air mixture. Kakutkina et al.^39^ found that for a 70% hydrogen mixture, the flame propagates upstream at a distance of 100 mm in 2000 s, and the maximum temperature recorded was around 950 K at approximately 1750 s under steady-state conditions. Fuel interchangeability was studied by Alavandi and Agrawal^40^ by combusting lean blends of hydrogen-syngas and methane fuel mixtures inside a porous burner. The air flow rate was kept constant for all tests, while the methane concentration was lowered for each test as the hydrogen and carbon monoxide fuel rates were adjusted to produce the required thermal power under steady-state conditions. The authors^40^ reported reduced carbon monoxide and nitrogen oxide emissions at any flame temperature for hydrogen and carbon monoxide mixtures compared to those produced by a pure methane flame.

Gauthier et al.^41^ studied pollutant emissions when hydrogen was added to a natural gas mixture inside a porous burner. The burner was first operated with a natural gas–air mixture; once the combustion was stabilized, a gradual addition of hydrogen was made. The experiments were conducted for 0.3 ≤ ϕ ≤ 0.95, 100 ≤ *P* ≤ 700 and an interchangeable hydrogen concentration of up to 100% within the natural-gas and hydrogen fuel composition. Gauthier et al.^41^ reported that as natural gas is slowly replaced by hydrogen a reduction in carbon monoxide, carbon dioxide, and nitrogen oxides took place. Also, they found that when the hydrogen content exceeds 80%, the flame becomes unstable.

Peng et al.^34^ experimentally examined the combustion of a premixed hydrogen–air mixture by varying the size of the combustion chamber inside the porous burner. The purpose of the study was to monitor the flame stability with the inclusion and exclusion of porous media in the combustion chamber. The authors^34^ chose a stainless-steel mesh to represent the porous media, whereby the hydrogen–air mixture was operated for different mass flow rates and equivalence ratios. Peng et al.^34^ found that as the diameter of combustor chamber diminished, the flame front within the porous media enlarged across the flow direction. Also, with the insertion of porous media, an increase in heat transfer accelerated the combustion process. Inspired by Alavandi and Agrawal,^40^ Arrieta et al.^42^ studied the combustion of mixtures of methane and syngas inside a porous burner. Their experiments were focused on the emissions of CO and NO_x_, flame stability response to assigned thermal power, and the effects of volume fraction of the syngas mixtures under steady-state conditions. Arrieta et al.^42^ used methane as the basic fuel for all tests where the hydrogen to carbon monoxide ratio was varied. It was concluded that the addition of hydrogen-rich syngas to methane did not impact the flame stability or temperature profile drastically. Yet, a considerable reduction in CO and NO_x_ emissions was reported.

The most recent work of the current authors showed that an ultralean mixture of methane and carbon dioxide burning inside a porous foam could strongly respond to fluctuations in the methane flow rate.^38^ Upon introduction of fluctuations, the flame featured hydrodynamic motion inside the porous foam. It was observed that there were certain amplitude and frequency of flow modulation under which the flame could survive.^38^ As the characteristics of CH_4_–H_2_ blends are quite distinctive to those of CH_4_–CO_2_, the burner stability for combustion of methane and hydrogen blends needs to be re-examined. This is particularly due to the high reactivity of hydrogen which can significantly affect flame blow-off and extinction.

As heat transfer dominates combustion in porous media, the latter is expected to be influenced by variations in the inlet flow. This is particularly the case in ultralean combustion in which changes in the temperature can have a profound effect on the flame stability. Nevertheless, as seen in the preceding survey of literature, currently, there is no systematic experimental study on CH_4_–H_2_ mixtures subject to inlet flow disturbances under ultralean conditions. In an attempt to address this shortcoming, the current work investigates experimentally the unsteady ultralean hydrogen combustion of different blends of methane and hydrogen.

## 2 Methodology

### 2.1 Experimental Setup and Instrumentation

**Figure 1:**
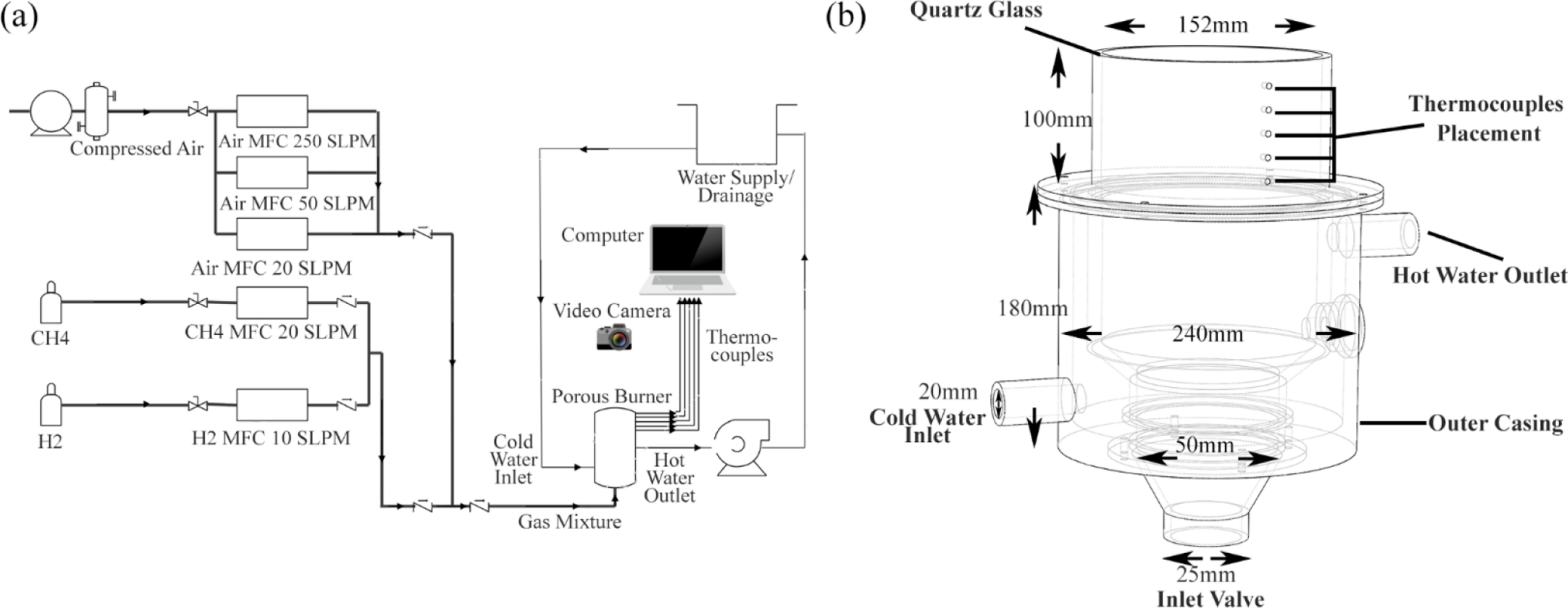
(a) Diagrammatic representation of the experimental setup. (b) 3D transparent view of the employed porous burner.

A schematic representation of the experimental setup is illustrated in Figure 1a. This consists of four key components; the porous burner (Figure 1b), water coolant system, fuel mixture supply, and data harvesting and measurement apparatus.

#### 2.1.1 Porous Burner

Figure 1b illustrates the vital sizes of the porous burner in a transparent 3-D model. The model was made up of four main parts consisting of a quartz glass tube, an inlet valve, a combustion compartment, and an outer casing. The flame in porous media was observed through the quartz glass tube by securing it above the burner with a sealant able to withstand temperatures up to 1500 K. The remaining parts were made of stainless steel.

**Figure 2:**
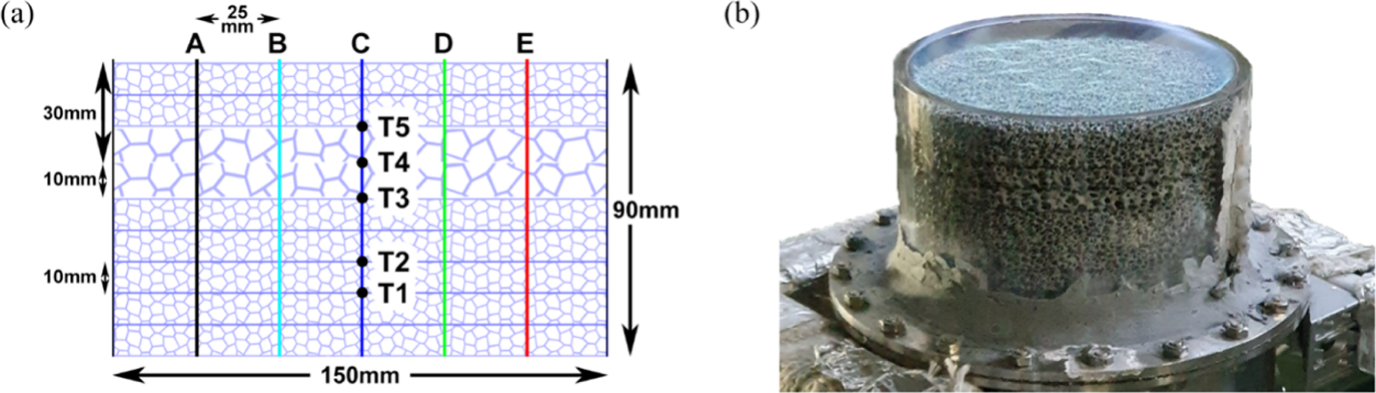
(a) Combustion region schematic highlighting the placement of thermocouples with reference points A, B, C, D, and E. (b) Quartz glass tube visualizing the flame before entering the porous foam.

The combustion compartment was made up of two separate sections: the combustion and preheating regions. Both regions consisted of porous ceramic layers stacked one atop the other vertically. The preheating section (designed to reduce the probability of flashback) entailed an Al_2_O_3_ foam (20 ppi—ε ≈ 0.47) positioned nearest to the inlet valve preceded by a funnel-shaped SiC ceramic foam (20 ppi—ε ≈ 0.47). This was followed by a stack of low-density SiC foams (10 ppi—ε ≈ 0.72) completing the preheating region. The combustion region (visible through the quartz glass tube) consisted of predominately high-density SiC foams (20 ppi—ε ≈ 0.47). Two layers of low-density SiC foams (10 ppi—ε ≈ 0.72) were placed near the center of the combustion region to provide further flame stability as illustrated in Figure 2.

#### 2.1.2 Water Coolant System

An opening was created between the internal side of the outer casing and the external side of the combustion compartment for water cooling to take place. The vacant gap acted as a water reservoir with an integrated inlet and outlet within the porous burner. The water coolant system drew cool water from the water source filling the water reservoir from the inlet located near the bottom of the burner. Once the reservoir was full, the water departed from the outlet (located near the top of the burner) warranting a thorough cooling of the stainless-steel burner.

#### 2.1.3 Fuel Mixture Supply

The mixture entering the burner consisted of a blend of hydrogen and methane diluted with air. Fuel was supplied from the corresponding gas cylinders and digital mass flow controllers (MFCs) equipped with manually operated valves. Moisture was removed from the compressed air with the use of an air filter, and the air was supplied in an arrangement similar to that of the fuel where a quarter-turn hand valve was installed before the air MFCs. Flow Vision SC software was obtained from ALICAT to vary the mass flow rate of each MFC using a computer. The programmable MFCs from ALICAT Scientific operated under an uncertainty of ±0.6% for both air and fuel. A number of MFCs were installed with relevant operational ranges (Figure 1a) for fuel and air to operate under steady and time-varying flows. The fuel mixture was premixed within the transmission system before being supplied to the porous burner. Air was supplied via a rubber pipe with an outer diameter of 25.4 mm, which was connected to a 6.35 mm diameter stainless steel pipe carrying fuel further downstream where the premixing occurred. The rubber pipe fed the fuel mixture to the inlet valve of the porous burner.

#### 2.1.4 Data Harvesting

Figure 2a depicts the five key points wherein temperature measurements were conducted at the midpoint of the ceramic foam. Five respective holes were drilled on the side of the quartz tube glass to secure each thermocouple midpoint of the porous media with the use of a high-temperature resistant sealant. To measure flame temperatures, a type N thermocouple was deployed with a uncertainty error of ±2.5 K, minute diameter size of 0.5 mm, and the capability of withstanding temperatures up to 1553 K. Pico software was utilized to plot temperature with respect to time by translating the voltage signals produced by the thermocouples. In order to capture the temperature change accurately, data were logged at much larger frequency (100 Hz) than the set frequency of the inlet fuel disturbances (≈0.02 Hz). The exhaust gases of the porous burner were monitored by installing a gas analyzer—Anton Sprint Pro 5—above the outlet of the burner (≈10 cm) for cases described in Table 1. The error was stated to be ±0.3% for CO_2_ and ±20 ppm for CO. A full high-definition video camera (1920 × 1080) was mounted ≈1.5 m from the burner to record the flame performance and migration.

**Table 1:** Steady Experiments

			a-CH_4_(90%)–H_2_(10%)		b-CH_4_(70%)–H_2_(30%)	
	H_2_ (standard L/min)	CH_4_ (standard L/min)	air (standard L/min)	mixture (standard L/min)	equivalence ratio (ϕ)	thermal power (kW)
case 1a	0.57	5.16	190.86	196.59	0.25	2.5
case 2a	0.69	6.24	209.94	216.87	0.275	3.02
case 3a	0.76	6.81	229.03	236.6	0.275	3.3
case 4a	0.82	7.37	248.12	256.31	0.275	3.575
case 1b	2.11	4.91	178.03	185.05	0.275	2.47
case 2b	2.34	5.46	197.81	205.61	0.275	2.75

### 2.2 Experimental Procedure

The equivalence ratio^43^ was noted to be
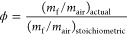
1where the mixture is described as lean when ϕ < 1. The thermal power of the system^43,44^ is given by
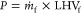
2in which the energy produced by each fuel is determined.

Table 1 presents the operating conditions for steady experiments, and Table 2 shows those for the unsteady experiments, for varying fuel concentrations of methane and hydrogen mixtures. The mixture blends consist of a mole percentage of each respective fuel. The flow rates of both fuels were systematically altered by programming the MFCs using Flow Vision SC software. Under lean operation (ϕ ≈ 0.55), the porous burner was ignited using a long-nosed lighter. The mixture flow rate was then gradually lowered to achieve ultralean combustion (0.25 ≤ ϕ ≤ 0.275) and flame stability within the porous media. A gas analyzer (Anton Sprint Pro 5) was employed to record the final values of CO, CO_2_, and NO_x_ of the cases described in Table 1 after flame stability had been achieved. It was found that NO_x_ emissions are almost negligible. This is to be expected as the current low-temperature combustion system strongly suppresses formation of thermal NO_x_. Once the flame had been stabilized, the concluding temperature values were noted from Pico software.

**Table 2:** Oscillatory Experiments

			x-CH_4_(90%)–H_2_(10%)			y-CH_4_(70%)–H_2_(30%)		
	H_2_ (standard L/m)	CH_4_ (standard L/m)	air (standard L/m)	mixture (standard L/m)	equivalence ratio (ϕ)	thermal power (kW)	oscillation period (s) of fuel flow	amplitude (%) of steady fuel flow
case 1x	0.82	6.63–8.11	248.1	255.57–257.05	0.2481–0.3016	3.221–3.926	60 s	CH_4_ = 10%
case 2x	0.82	5.16–9.58	248.1	254.10–258.52	0.1945–0.3550	2.514–4.629	60 s	CH_4_ = 30%
case 3x	0.82	3.69–11.06	248.1	252.63–260	0.1413–0.4088	1.8130–5.3380	60 s	CH_4_ = 50%
case 4x	0.74–0.9	7.37	248.1	256.23–256.39	0.2743–0.2755	3.571–3.577	60 s	H_2_ = 10%
case 5x	0.57–1.09	7.37	248.1	256.04–256.54	0.2725–0.2770	3.556–3.587	60 s	H_2_ = 30%
case 6x	0.41–1.23	7.37	248.1	255.88–256.7	0.2712–0.2780	3.549–3.593	60 s	H_2_ = 50%
case 1y	2.11	4.42–5.4	178.03	184.56–185.54	0.25–0.3	2.238–2.711	60 s	CH_4_ = 10%
case 2y	2.11	3.44–6.38	178.03	183.58–186.52	0.2005–0.3490	1.770–3.1750	60 s	CH_4_ = 30%
case 3y	2.11	2.46–7.37	178.03	182.60–187.51	0.1508–0.3992	1.3–3.649	60 s	CH_4_ = 50%
case 4y	1.90–2.32	4.91	178.03	184.84–185.26	0.2722–0.2774	2.4610–2.4850	60 s	H_2_ = 10%
case 5y	1.48–2.74	4.91	178.03	184.42–185.68	0.2672–0.2830	2.438–2.513	60 s	H_2_ = 30%
case 6y	1.06–3.17	4.91	178.03	184–186.11	0.2615–0.2882	2.41–2.537	60 s	H_2_ = 50%

After the flame was stabilized, as described in Table 2, the hydrogen and methane flows were subject to a sinusoid disturbance with a varying amplitude of 10–50% of its initial value for both fuel-concentrated mixtures with a single forcing frequency. This was conducted by programming the MFCs to oscillate the fuel flow rates at the designated amplitude and frequency. As the flame inlet disturbance was introduced, the temperature traces were recorded using the Pico software at the thermocouple locations illustrated in Figure 2a. The digital camera recorded the flame movement, which was then reported along with the designated reference points (Figure 2a) with an image processing code developed in MATLAB.

Uncertainty error was also calculated to ensure adequate accuracy of the experimental procedure during flow modulation. This included the uncertainty error for MFCs and thermocouples. To determine this, thermocouple values were used from case 6x where T3 reported the highest temperature (1384 K). Therefore, the T3 uncertainty error was assumed across all five thermocouples. Combined with the supplied MFC manufacturer error (±0.6%), the total accumulative uncertainty error in flow measurements = ±2.7%.

### 2.3 Image Processing

An in-depth image analysis was carried out to observe the change in flame position. The video recording of an entire oscillation of 60 s was cut out from the footage of each case described in Table 2. Using video editing software Adobe Premier Pro, screenshots of the oscillation were saved every 2 s from the video recordings of all cases to examine the flame operation. Additionally, an image processing code was developed in MATLAB to examine the screenshots. Each screenshot was initially cropped to represent only the ceramic foam within the quartz tube glass (see Figure 2a). To monitor flame movement, the screenshot pixels were converted to a distance where 12.8 pixels were noted to be an equivalent of 1 mm. The referenced points were generated vertically along the *x*-axis to determine the flame migration in the *y*-direction. Every picture was transformed from an RGB image to black and white where a brightness criterion was introduced for flame detection. The luminosity value for each concentration of fuel varied thus authenticated by visual validation. As the brightness conditions were fulfilled, the vertical position of the parameter was determined (i.e. where the flame had been identified) at the specified reference points. The designated values were reduced by filtering out only the top and bottom *y*-location. These values served to define the upper and lower section of the flame across all reference points. This procedure reoccurred until all pictures had been processed by the code. The final top and bottom positions of the brightness criterion were then plotted with respect to time, illustrating migration of the flame vertically within the porous media along the five reference points over a full oscillatory cycle.

## 3 Results and Discussion

The burner was operated under a steady state as well as fluctuating fuel flow. Here, the outcomes of these two modes of operation are presented separately.

### 3.1 Steady-state Conditions

**Figure 3:**
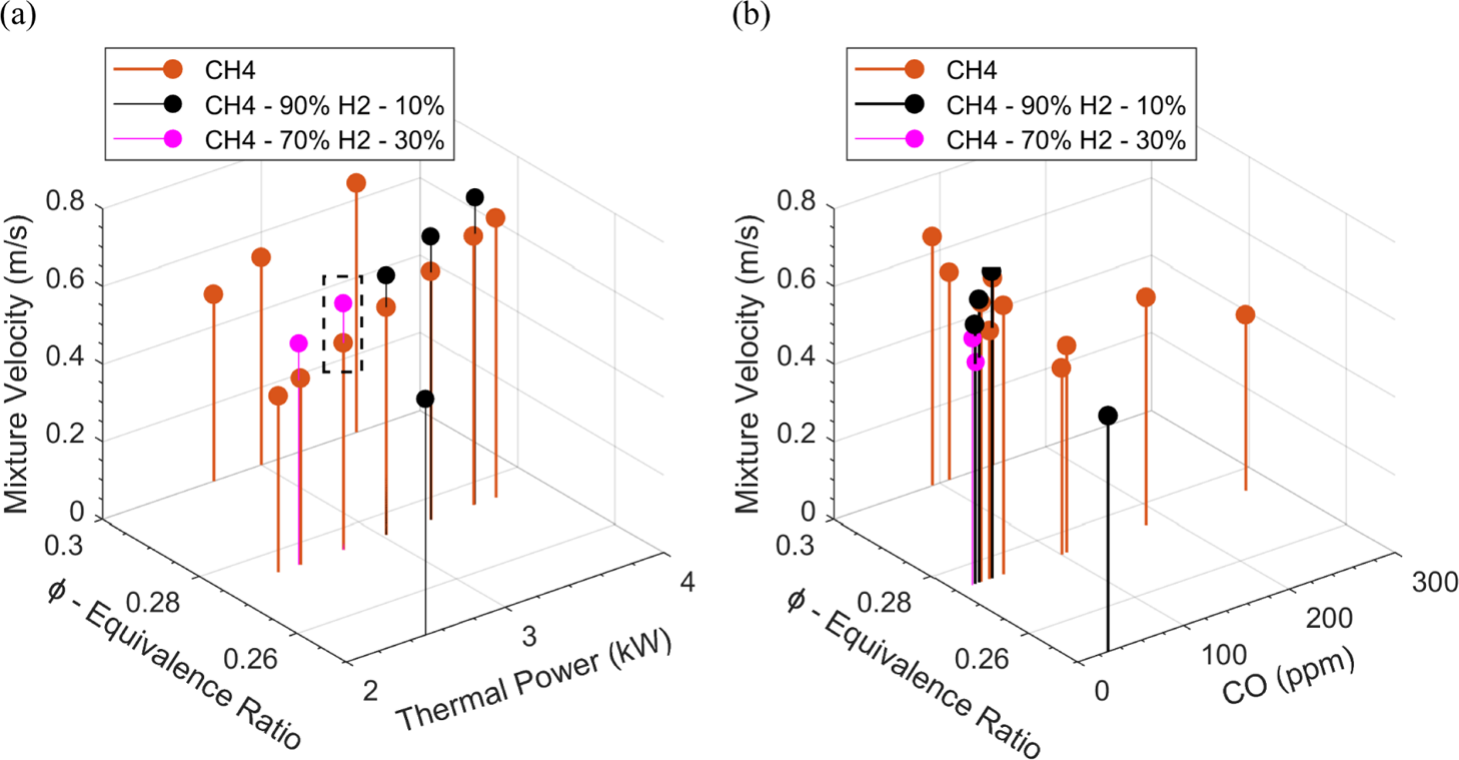
Steady fuel mixtures. (a) ϕ vs *P*. (b) ϕ vs CO emissions.

In order to achieve stable combustion inside the ceramic foam, a fine balance between heat production, heat deficit, and heat recirculation is required.^18^ Flame stabilization is most commonly attained when the flame speed matches the upstream flow velocity of air and fuel mixture.^27^Figure 3 depicts the effectiveness of stable combustion within the porous burner functioning in an ultralean setting for various fuel mixtures. Habib et al.^38^ have extensively discussed the response of methane and biogas mixtures within the burner shown in Figure 1. Therefore, the focus of the current discussion will be primarily on methane and hydrogen mixtures (Table 1). Figure 3a displays the correlation between mixture velocity, equivalence ratio, and thermal power. The unburned mixture velocity can be considered as the filter velocity within the quartz tube upstream of the flame. Noticeably, there exists a uniform pattern among all fuel mixtures where the thermal power augments constantly as the mixture velocity is increased. It is also clear that the CH_4_(90%) + H_2_(10%) mixture exhibits a larger thermal power when compared to its counterpart CH_4_(70%) + H_2_(30%); which is predominantly due to its larger concentration of methane as (per unit volume) methane has a higher enthalpy of combustion compared to hydrogen. Further, CH_4_(90%) + H_2_(10%) is able to operate at ϕ = 0.25 as hydrogen provides a higher flame temperature than methane alone to preheat the incoming cold reactants and avoid flame extinction.

Figure 3b illustrates the impact of the equivalence ratio and mixture velocity on carbon monoxide emissions. An apparent pattern is visible where the carbon monoxide emissions drop as the mixture velocity is increased and the equivalence ratio is decreased. The temperature in the combustion region plummets when mixture velocity curtails. This is typically due to a decline in heat generation, whereas the loss of heat continues uninterruptedly. The reduced temperature increases the risk of incomplete combustion and retards the process of oxidation of carbon monoxide to carbon dioxide. Carbon monoxide emissions also increase at smaller equivalence ratios as it chokes the heat release and reduces the reaction temperature.

**Figure 4:**
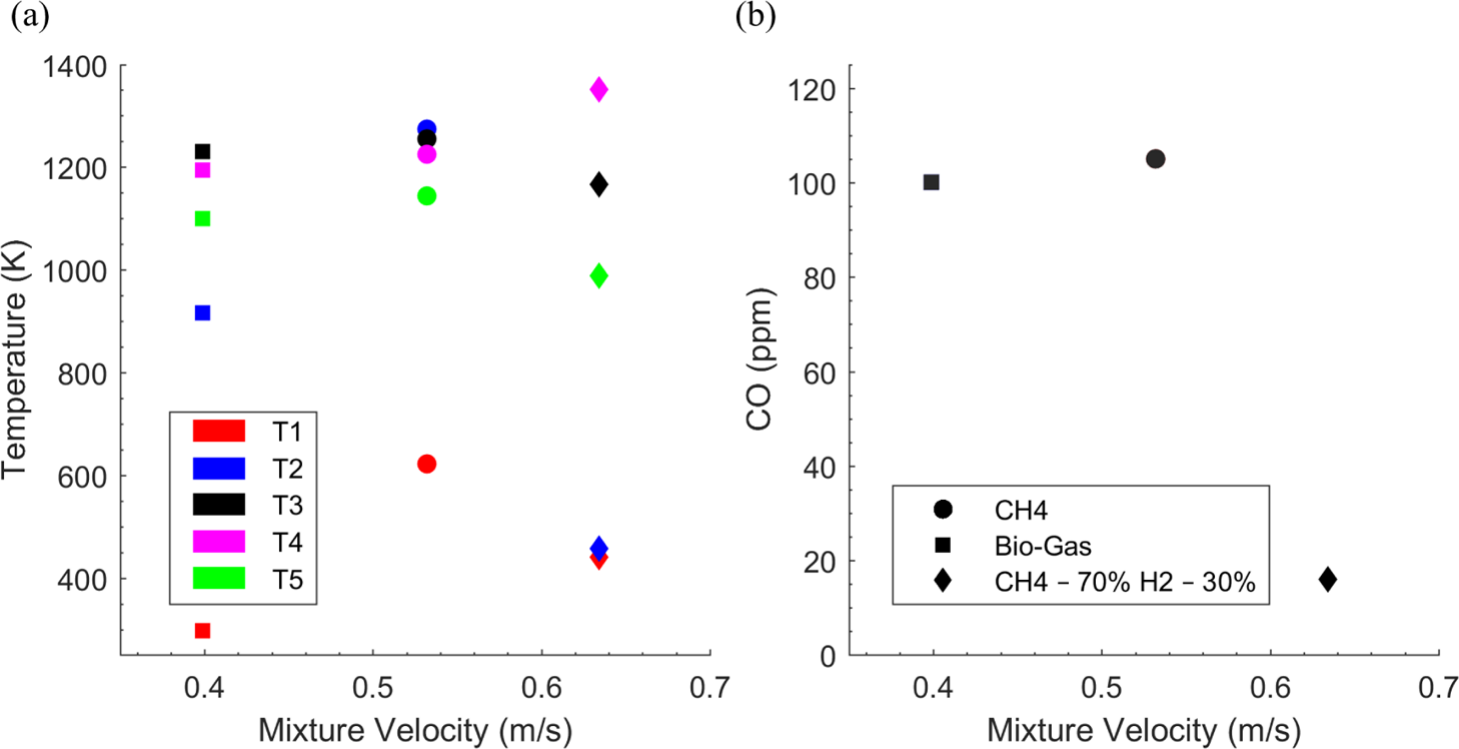
Temperatures and CO production vs mixture flow velocity under steady state conditions (no modulation of the fuel stream). CH_4_—(circle), biogas (CH_4_(70%)–CO_2_(30%))—(square), CH_4_(70%)–H_2_(30%)—(diamond), ϕ = 0.275. (a) mixture velocity vs temperature. (b) Mixture velocity vs CO emissions.

Figure 4 depicts the temperature and carbon monoxide emissions plotted with mixture velocity. The three fuel mixtures operate on an identical equivalence ratio of 0.275 and produce the same thermal power of 2.75 kW. Figure 4a shows the five thermocouple readings when stable combustion has been achieved for the selected fuel mixtures. It is evident that higher unburned mixture velocities result in the larger temperatures. The fuel composition also strongly affects the temperature where biogas outputs the lowest temperature for similar operating conditions. This occurs as a response to the initial presence of carbon dioxide, which dilutes the fuel mixture and lowers the flame temperature. Further, the CH_4_(70%)–H_2_(30%) mixture produces the highest temperature (1340 K), followed by CH_4_ (1291 K) and then biogas (1233 K). This is due to the addition of hydrogen to methane, as hydrogen produces a greater flame temperature when compared to pure methane. It is also noted that the peak temperature for each mixture is detected at a different thermocouple point as the flame location within the burner differs between the fuel mixtures. In Figure 4b, the carbon monoxide emissions follow a similar trend as Figure 3b, where a greater mixture velocity produces lower emissions. In addition to mixture velocity, hydrogen plays a key role in reducing the carbon monoxide emission for the CH_4_(70%)–H_2_(30%) mixture. The CH_4_(70%)–H_2_(30%) mixture shows 85% reduction in carbon monoxide emissions in contrast to CH_4_. This could be primarily attributed to the higher flame temperatures of CH_4_–H_2_ blends compared to those of CH_4_ and biogas. High temperature facilitates the oxidation of carbon monoxide into carbon dioxide and thus reduces CO emissions.

### 3.2 Fluctuating Fuel Flow

**Figure 5:**
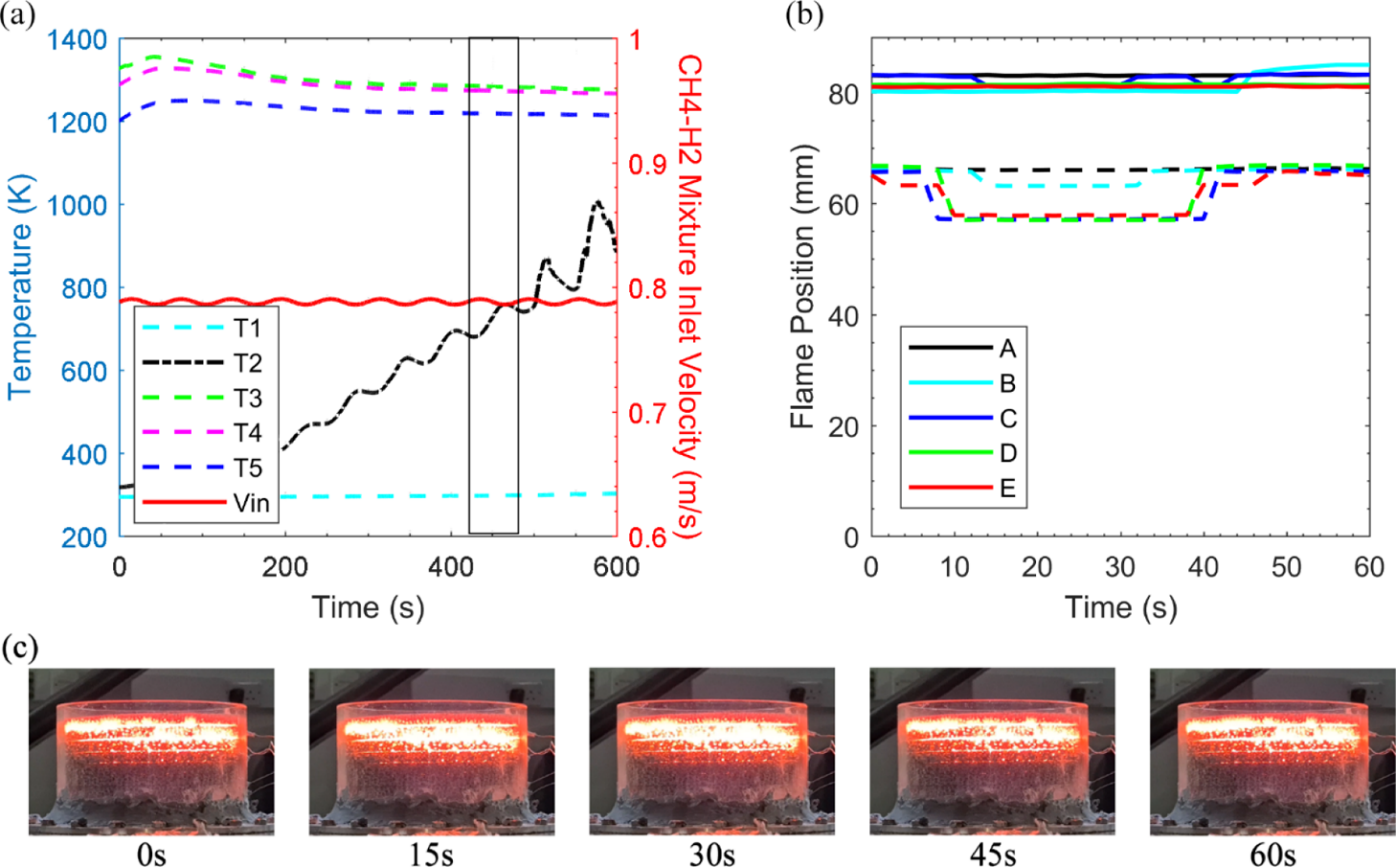
Forced response of the burner to modulation of fuel streams. Case 1x, amplitude of oscillation in methane flow: 10%. (a) Temperature + CH_4_(90%)–H_2_(10%) mixture velocity vs time. (b) Flame position motion at reference points, top section of the flame (-), bottom section of the flame (- -). (c) Screenshots of burner responding to oscillatory flow during complete cycle.

Case 4a was incorporated as a foundation for unsteady CH_4_(90%)–H_2_(10%) cases before superimposing oscillatory disturbances at the inlet. Figure 5 displays the porous burner operation with the CH_4_(90%)–H_2_(10%) mixture subject to an oscillatory disturbance introduced on the methane flow with an amplitude of 10% of its steady value, over a period of 60 s. The thermocouples were used to record the temperature of each case where the cut-off temperature to detect flashback was defined as *T* = 773.15 K. The temporal variation of methane flow leads to a distinct movement of the reaction region within the ceramic foam. This flame movement was video recorded (Section 2.2), and the change in temperature was also recorded. Ten cycles of fuel flow modulation was applied, while no flame flashback/blow-off was observed.

Figure 5a illustrates the variation in temperature for case 1x, including the mixture velocity trace. Evidently, the third thermocouple displays the highest temperature recorded (1354 K) in between the varying density of the SiC foams. This boundary surrounded by the two SiC foams grants additional flame stability during burner operation as the high-density SiC foam operates as a flashback arrestor. Further, the thermocouples report predominately fixed temperatures in response to the oscillatory methane fuel apart from the second thermocouple. This indicates that the flame movement was not detected by the temperature measurement apparatus. For the current experiment, this implies that the flame movement being restricted around the location of two adjacent thermocouples. This phenomenon was also reported for CH_4_ and biogas by Habib et al.^38^ As a complimentary analysis, Figure 5b depicts the change in location of the flame fluctuation during the eighth oscillatory cycle (marked by the box in Figure 5a) at the nominated *y*-direction reference points. Approximately 8 s later, the bottom section of the flame expands about 7 mm upstream as shown by Figure 5c (15 s, 30 s). The flame expansion ties in with the temperature measurement noted at the second thermocouple. Figure 5b visualizes the flame motion to be roughly 7 mm, thus elucidating why the flame movement went undetected by the other thermocouples. An assessment amid the mixture velocity trace highlighted in Figure 5a, and the flame region in Figure 5b discloses that the flame movement almost accurately trails the changes in the flow velocity. Clearly, a phase lag exists among the inlet fuel flow and the highlighted temperature variation. This is to be expected, and the time lag between fuel and flame fluctuations has been already reported in the context of flame dynamics.^45^

**Figure 6:**
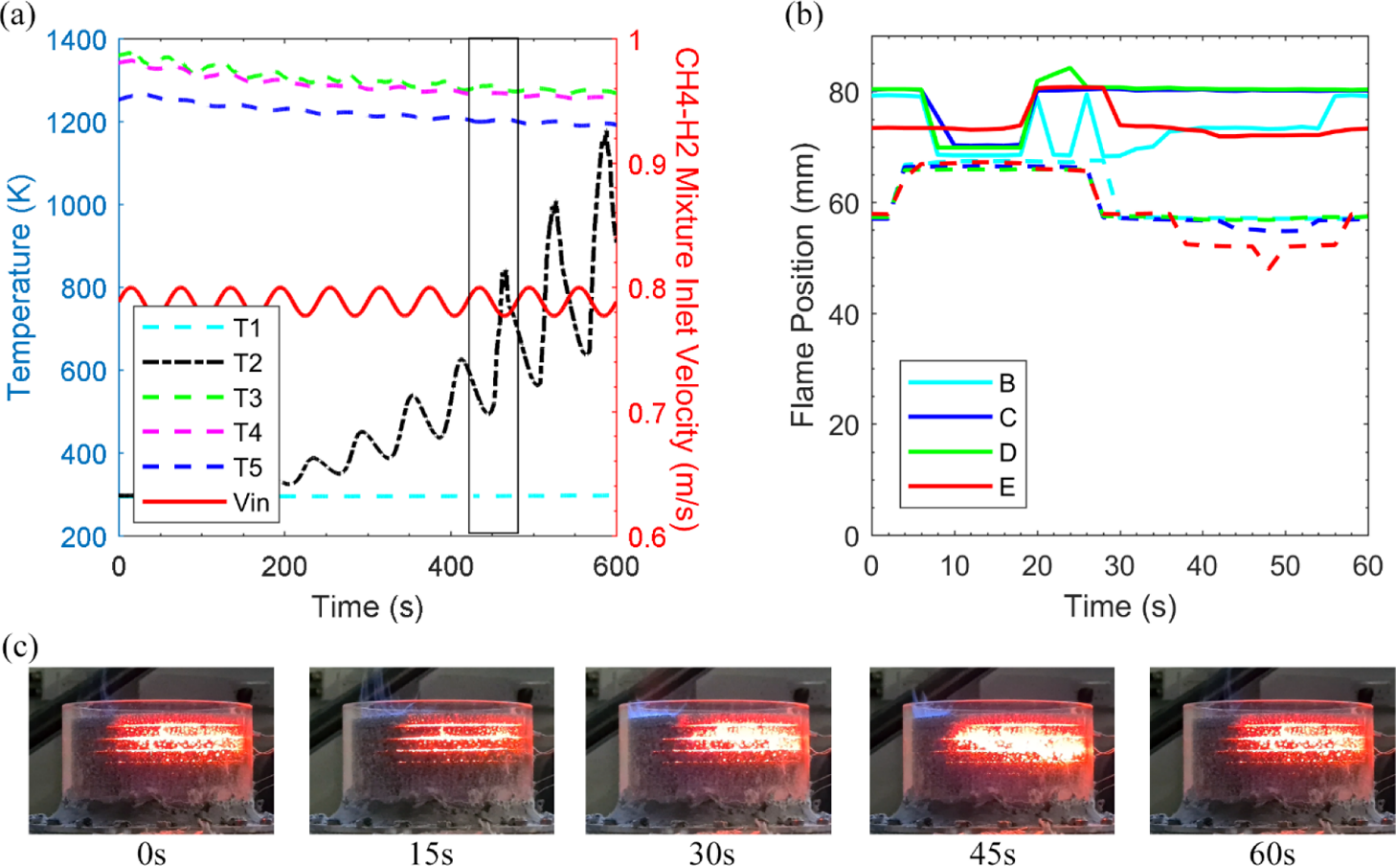
Forced response of the burner to modulation of fuel streams. Case 3x, amplitude of oscillation in methane flow: 50%. (a) Temperature + CH_4_(90%)–H_2_(10%) mixture velocity vs time. (b) Flame position motion at reference points, top section of the flame (-), bottom section of the flame (- -). (c) Screenshots of the burner responding to oscillatory flow during the complete cycle.

Figure 6 displays case 3x where the amplitude has been enlarged to 50% of the methane flow rate with the fluctuation period remaining unchanged. The temperature trace of the second thermocouple in Figure 6a records successive gains for the entirety of the experiment. However, in reality, the overall system is experiencing greater heat loss than heat generation. Consequently, if the present case was to continue for further methane oscillatory cycles, the flame would inevitably extinct. The alarm of blow-off is raised in Figure 6b, as no flame is found within the brightness criterion at reference point A, at which a slow process of flame extinction is ongoing. This process remains undetected by the thermocouples due to their central placement within the ceramic foams. Additionally, modulation of methane flow has a direct impact on the concentration of hydrogen and methane, while it also alters the equivalence ratio of the CH_4_(90%)–H_2_(10%) mixture. As a result, during the trough at an amplitude of 50% of methane oscillation, the equivalence ratio plummets to 0.1413. Due to this drop, the porous burner struggles to retain heat even after supply of the excess methane to the combustion chamber. A similar phenomenon was also reported by Habib et al.^38^ while operating the same burner on biogas. Yet, it was observed that an amplitude of 30% of the fuel flow rate for biogas was sufficient to incur greater heat loss. Further, for case 3x, an overall temperature reduction is observed at thermocouples 3, 4, and 5 with time as the preheating region receives minimal quantities of heat from the combustion region. As this continuous cycle of heat loss progresses, the substantial reduction in temperature rules out the possibility of sustaining a flame within the pores of the ceramic foam. Yet, a thicker flame is visible as a result of greater forcing amplitude despite very little flame motion.^46^ The heat loss can be monitored by the reduction in flame visibility in Figure 6c. It should be noted that a similar response was found for case 2x.

**Figure 7:**
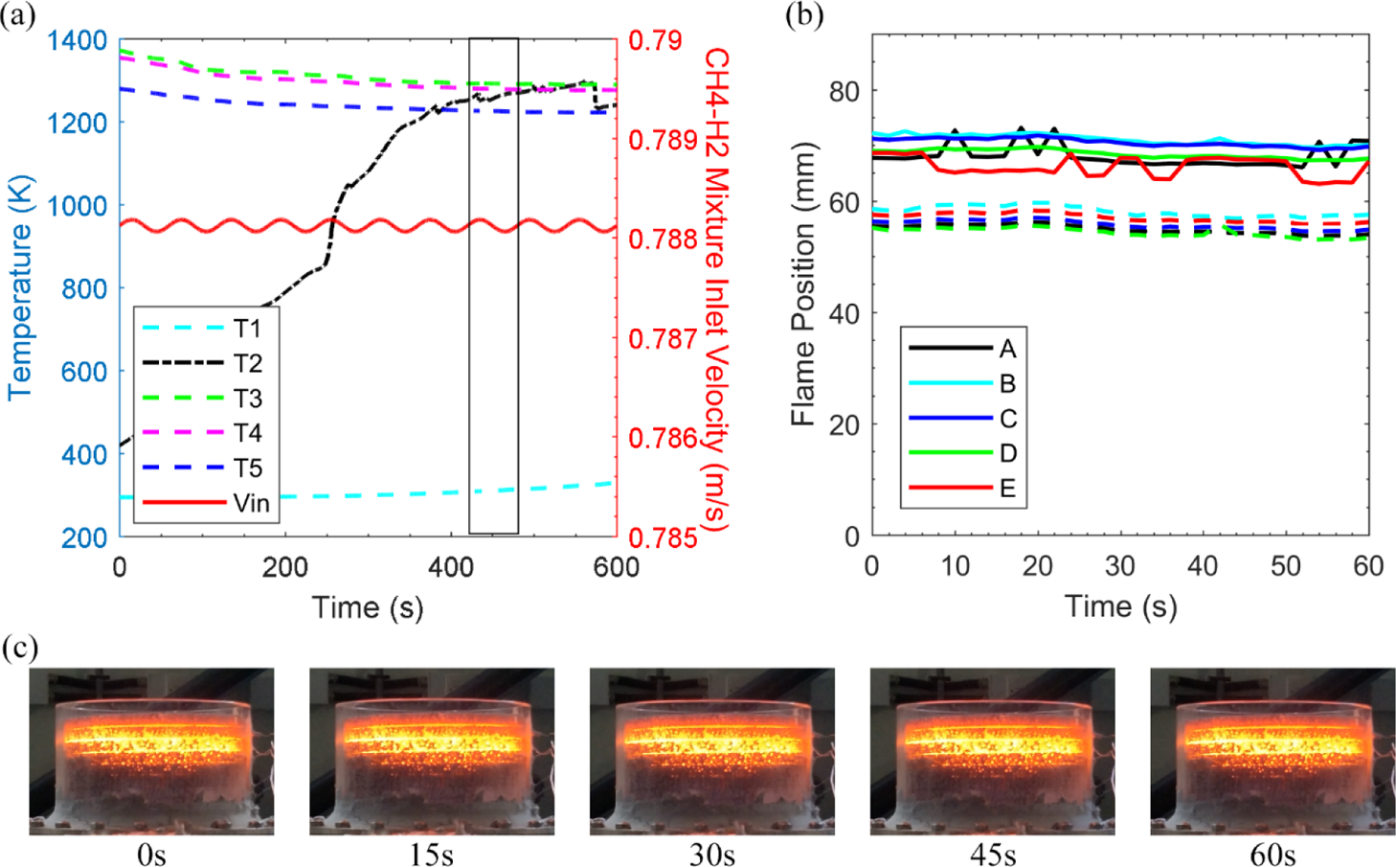
Forced response of the burner to modulation of fuel streams. Case 6x, amplitude of oscillation in hydrogen flow: 50%. (a) Temperature + CH_4_(90%)–H_2_(10%) mixture velocity vs time. (b) Flame position motion at reference points, top section of the flame (-), bottom section of the flame (- -). (c) Screenshots of the burner responding to oscillatory flow during a complete cycle.

Next, the fuel concentration of hydrogen in the CH_4_(90%)–H_2_(10%) mixture was subjected to inlet sinusoidal disturbances. Figure 7 displays case 6x where methane and air composition remain unchanged and the hydrogen component of the mixture undergoes oscillations at an amplitude of 50%. Case 6x was chosen for discussion due to its higher amplitude value as case 4x and 5x delivered a similar flame/temperature response. Because of the initial low concentration of hydrogen (10%), there are very small changes in the CH_4_(90%)–H_2_(10%) mixture velocity even with the hydrogen amplitude set as high as 50%. Therefore, it is noted that with the exception of the second thermocouple, most thermocouples are insensitive to the oscillations in hydrogen flow with the third thermocouple recording the highest temperature (1384 K) early on in the experiment. It can be seen that the porous burner withstands the introduced inlet disturbances without any visible signs of flame flashback or blow-off. A distinct contrast in the temperature trace of the second thermocouple emerges between the flow modulation of methane and hydrogen. The temperature trace in Figure 7 not only obtains a greater temperature but does so in a shorter time. However, the temperature response does not show any clear sign of large oscillations when compared to methane cases. This phenomenon can be attributed to the greater adiabatic flame temperature of hydrogen and its wider flammability limits. Even though the change in fuel flow is minimal, as a larger quantity of hydrogen fuel is burnt, greater heat is produced. Inevitably, this reduces the time for the heat to be transferred while substantiating the increase in temperature. Also, due to the superior thermal properties of SiC foam, heat is retained when hydrogen concentration is decreased. Figure 7b confirms minor flame motion at the eighth cycle of the experiment as temperature changes are minimal near the end of the experiment, displaying the insignificant impact of hydrogen oscillation. Figure 7c depicts that the CH_4_(90%)–H_2_(10%) mixture shows insignificant flame motion during the eighth cycle. It is noted that in this case, the brightness and color of the flame are not as vivid as related to methane modulation due to hydrogen flame being nearly invisible.

**Figure 8:**
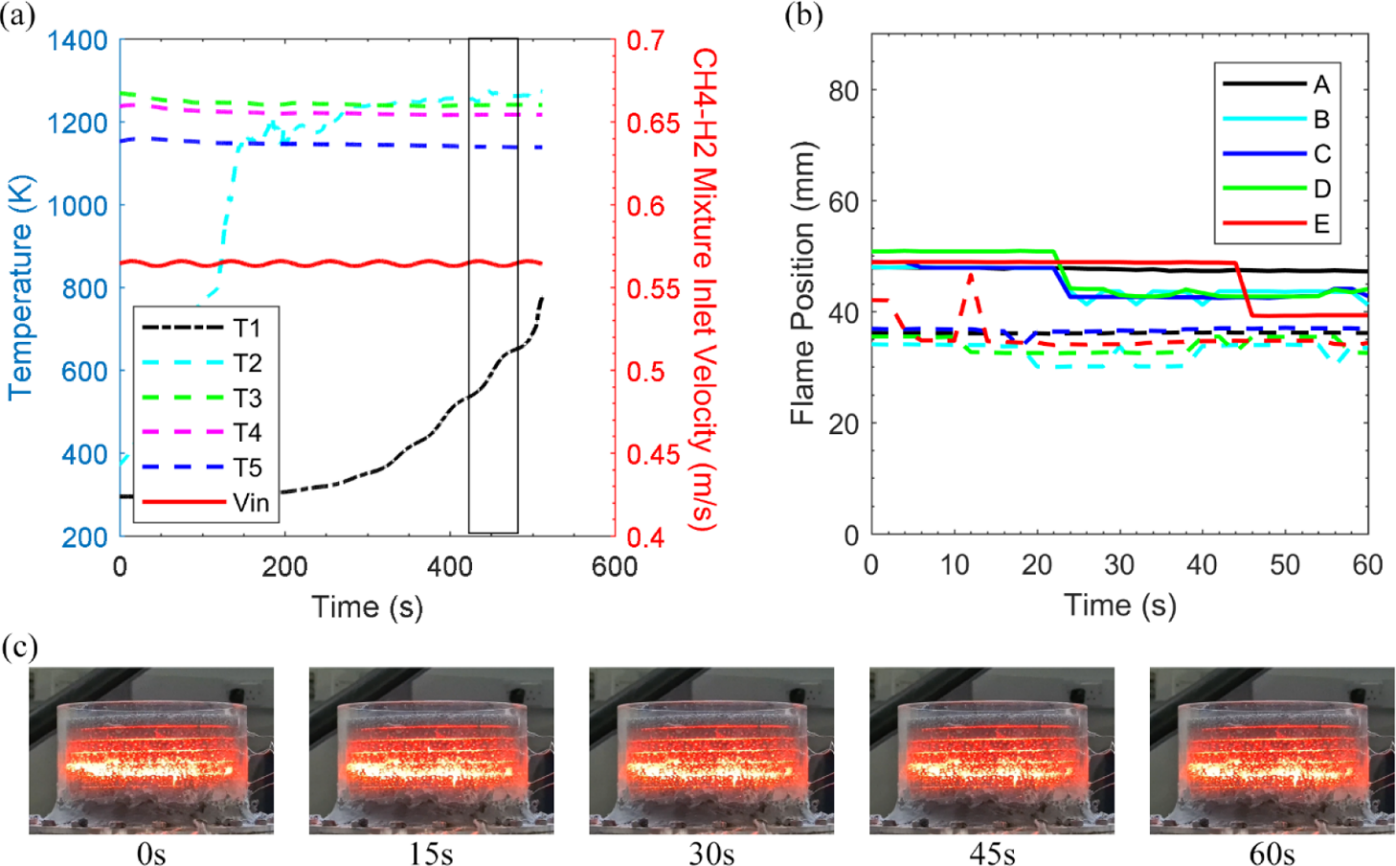
Forced response of the burner to modulation of fuel streams. Case 1y, amplitude of oscillation in methane flow: 10%. (a) Temperature + CH_4_(70%)–H_2_(30%) mixture velocity vs time. (b) Flame position motion at reference points, top section of the flame (-), bottom section of the flame (- -). (c) Screenshots of the burner responding to oscillatory flow during a complete cycle.

Figure 8 illustrates (case 1y) the temperature response and flame position of the CH_4_(70%)–H_2_(30%) mixture when methane flow is modulated with an amplitude of 10%. The starting point before introducing disturbances at the inlet was selected to be case 1b for additional safety. As the amplitude of oscillation is 10% for the methane flow rate, the equivalence ratio increases to 0.3 at the peak point of the oscillatory cycle, much greater than the value at which steady CH_4_(70%)–H_2_(30%) mixture operates stably. However, the local flame is choked when the equivalence ratio falls to 0.25 at the lowest point of the oscillatory cycle. Still, as the oscillatory cycle period is limited to 60 s, the system not only holds onto the heat but as the methane flow rate augments, the excess enthalpy combustion is fast-tracked. Consequently, in Figure 8a, during the ninth cycle, the fuel supply is cut as the flashback condition is met within 540 s. Flashback was also observed by Habib et al.^38^ as the same porous burner was operated solely on a CH_4_ mixture. However, despite the CH_4_ mixture having an identical amplitude of oscillation, it was noted that the flashback occurred due to a longer oscillation cycle (180 s). Figure 8b highlights the eighth oscillatory cycle of the flame motion. The flame motion appears to plummet along the respective reference points as the amplitude augments, with the flame size shrinking as much as 9 mm at point E. Figure 8c shows this occurrence as the flame not only moves further upstream but the width of the flame expands within the porous foam.

**Figure 9:**
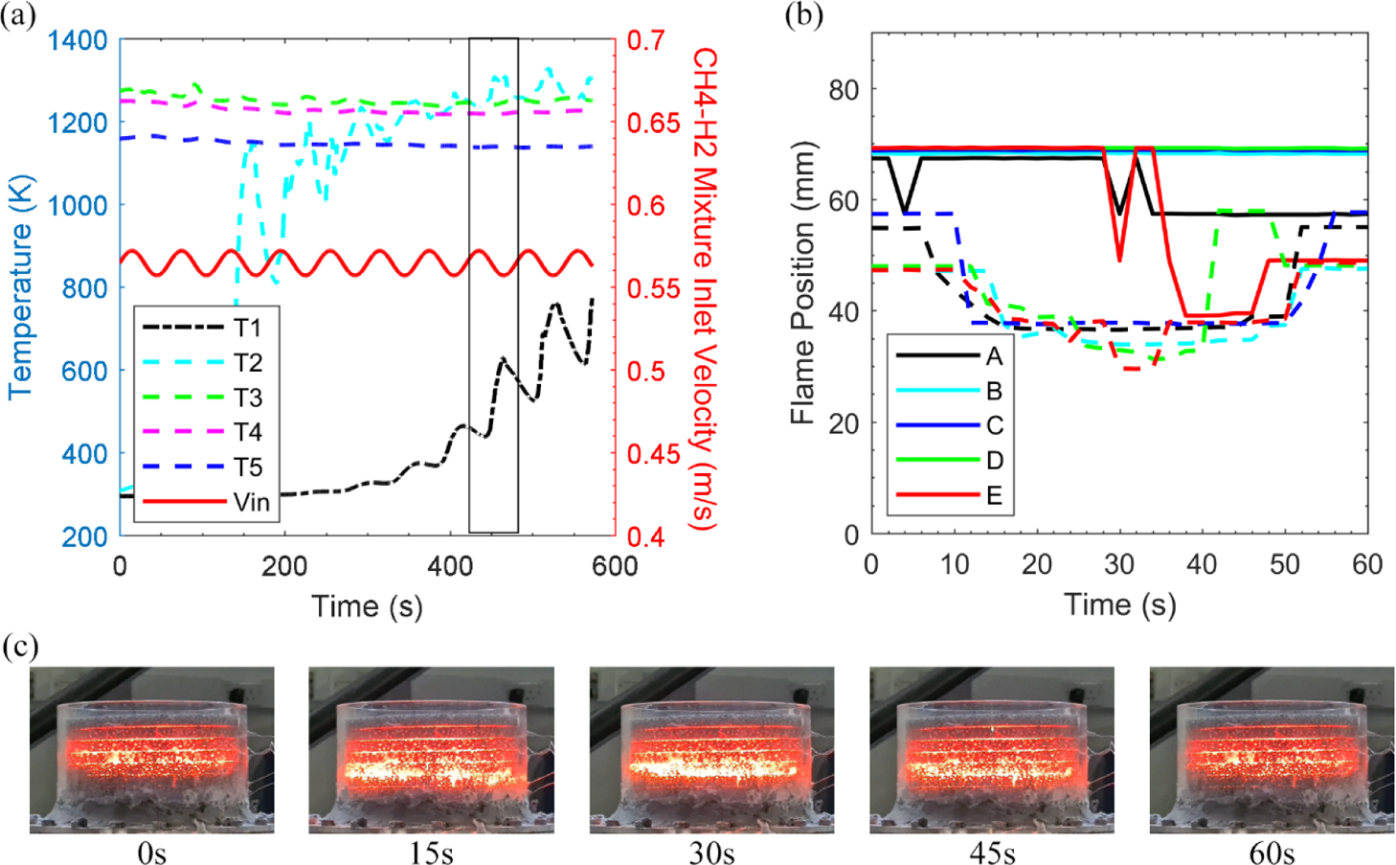
Forced response of the burner to modulation of fuel streams. Case 3y, amplitude of oscillation in methane flow: 50%. (a) Temperature + CH_4_(70%)–H_2_(30%) mixture velocity vs time. (b) Flame position motion at reference points, top section of the flame (-), bottom section of the flame (- -). (c) Screenshots of the burner responding to oscillatory flow during the complete cycle.

Figure 9 displays a parallel experiment to cases 1y and 2y except with a 50% amplitude. The temperature is primarily reported by the third thermocouple in Figure 9a. However, as the experiment proceeds, at the eighth cycle, the second thermocouple becomes the dominant respondent to the inlet fluctuations, followed by the first thermocouple. The recirculation of heat drastically improves as the amplitude of the methane inlet is augmented. This is because of the enhancement of heat convection at higher flow velocities. Downstream of the combustion region, there is a likelihood of a greater temperature at the lesser density porous foam as convective heat transfer takes place between the ceramic foam and the combustion products. Then, conductive and radiative heat transfer are further involved via the solid SiC foam upstream of the reaction region. As the foam presents a greater temperature than the CH_4_(70%)–H_2_(30%) mixture; convection amid the fluid and solid occurs and warms the cold reactants. Yet, due to the existence of the low porosity ceramic foam downstream of the combustion region, conduction is accelerated in response to significant methane fluctuations. Figure 9c highlights the flame motion whereby a somewhat sinusoid resemblance is observed relating to the temperature response of the porous burner.

**Figure 10:**
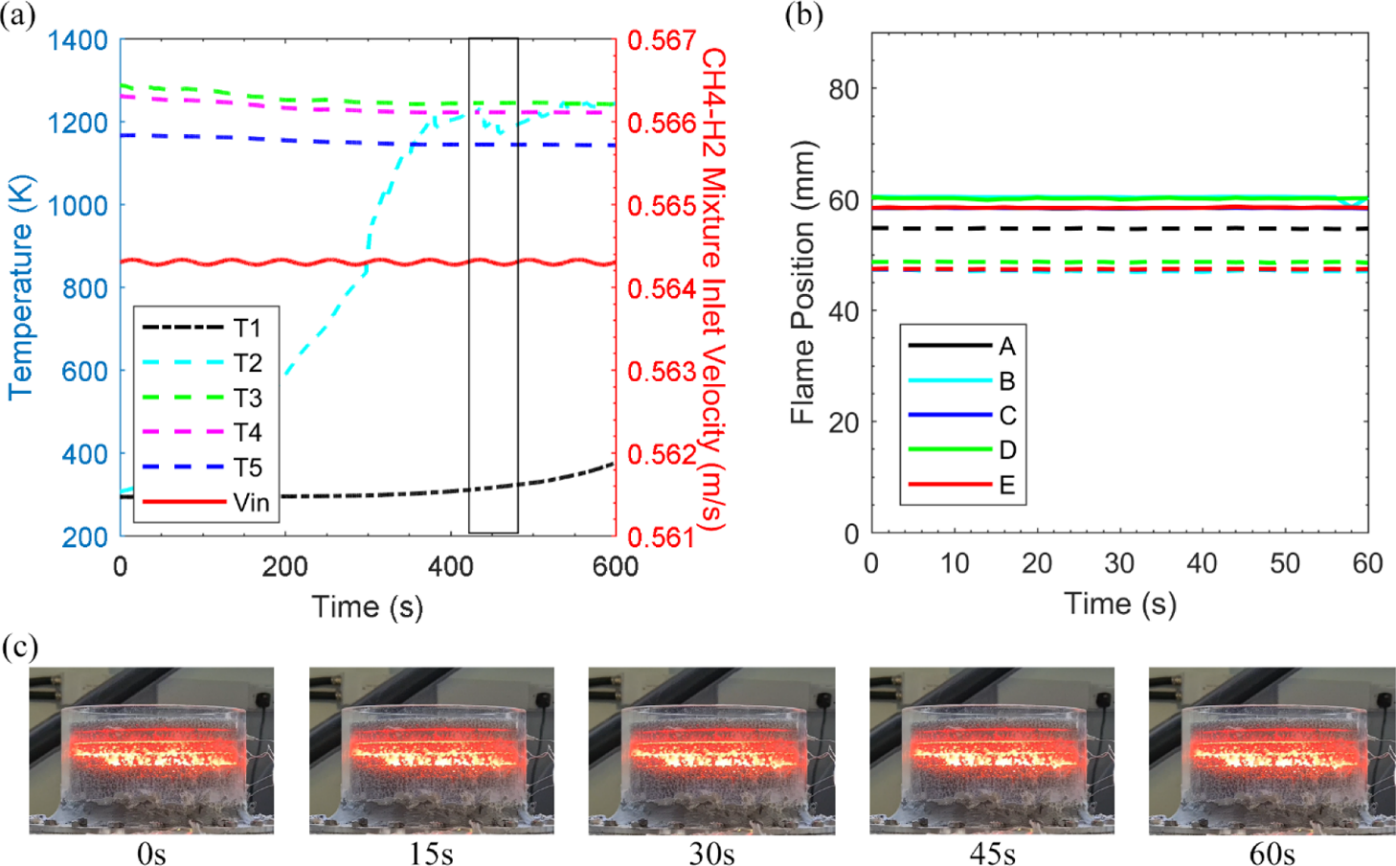
Forced response of the burner to modulation of fuel streams. Case 4y, amplitude of oscillation in hydrogen flow: 10%. (a) Temperature + CH_4_(70%)–H_2_(30%) mixture velocity vs time. (b) Flame position motion at reference points, top section of the flame (-), bottom section of the flame (- -). (c) Screenshots of the burner responding to oscillatory flow during the complete cycle.

Figure 10 shows the temperature response of the CH_4_(70%)–H_2_(30%) flame as the amplitude of hydrogen flow is modulated by 10%. Figure 10a illustrates a similar trend to that reported in case 6x, where the system remains stable throughout the experiment and does not distinctly follow the inlet oscillatory behavior of the fuel flow. The second thermocouple responds directly to the flame movement as it produces erratic behavior after the sixth sinusoidal wave. As this erratic response continues, a growth in the temperature is noted by the first thermocouple. Further, it is observed that due to the greater initial hydrogen concentration in the fuel composition, the thermal response of case 4y is similar to that of case 6x, even though the current experiment only superimposes an amplitude of 10% as compared to 50%. Figure 10a,b confirms that even when there is a greater portion of hydrogen present in the original fuel composition, a 10% fluctuation in amplitude has an insignificant impact on the flame motion.

**Figure 11:**
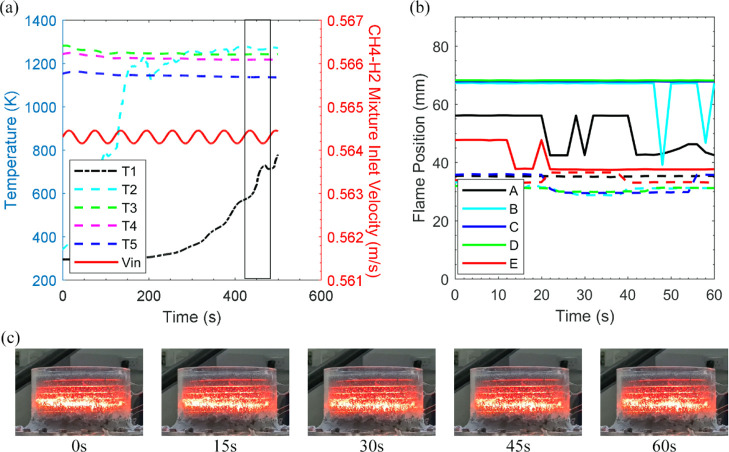
Forced response of the burner to modulation of fuel streams. Case 6y, amplitude of oscillation in hydrogen flow: 50%. (a) Temperature + CH_4_(70%)–H_2_(30%) mixture velocity vs time. (b) Flame position motion at reference points, top section of the flame (-), bottom section of the flame (- -). (c) Screenshots of the burner responding to oscillatory flow during the complete cycle.

Figure 11 illustrates the impact of imposing a 50% amplitude inlet disturbance on the hydrogen flow rate within the CH_4_(70%)–H_2_(30%) mixture. In this case, the porous burner manages to withstand around eight oscillatory cycles (Figure 11a) before undergoing flame flashback. It is noted that the thermal response transitions from the second to first thermocouple directly respond to the amplified sinusoidal disturbance at the inlet. Case 6x successfully mitigates the risks of flash back in contrast to case 6y for comparable operating conditions. Flashback occurs in case 6y due to a few reasons including the change in fuel composition and lower inlet mixture velocity, all correlating to the increase in the temperature. The greater portion of hydrogen increases heat transfer inside the SiC foam as a result of the larger temperature gradient amid the solid matrix and reacting gases. The flame motion during the eighth cycle is displayed in Figure 11b, showing a movement of more than 20 mm in some instances across the reference points. Subsequently, a wide flame front is detected. This is validated in Figure 11c, whereby not only the core flame width has been extended but also the external radiation glow has spread throughout the burner.

**Figure 12:**
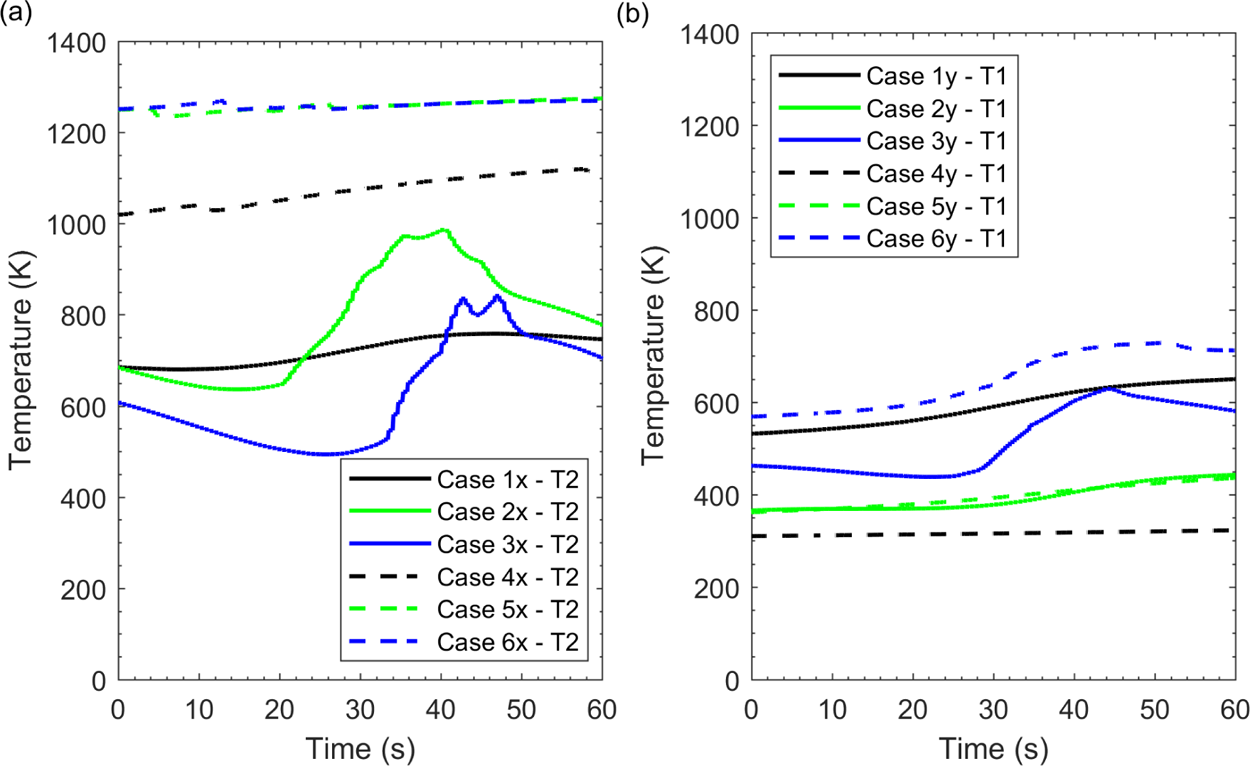
Forced response of the burner during an oscillatory cycle, CH_4_ modulation (solid line), and H_2_ modulation (dashed line). (a) CH_4_(90%)–H_2_(10%) T2 temperature vs time. (b) CH_4_(70%)–H_2_(30%) T1 temperature vs time.

Figure 12 presents a compilation of the temperature traces from the eighth oscillatory cycle of all cases from CH_4_(90%)–H_2_(10%) and CH_4_(70%)–H_2_(30%) mixtures. This comparison provides further clarity to the results reported earlier. Figure 12a clearly outlines case 3x undergoing blow-off as T2 records lower temperature against its counterparts—case 1x and case 2x—despite having a greater CH_4_ modulation amplitude. Similarly, H_2_ fuel modulation outputs larger temperature in comparison to CH_4_ modulation without directly responding to the oscillatory pattern. Case 5x and case 6x display a negligible temperature change. Figure 12b displays T1 traces of the CH_4_(70%)–H_2_(30%) mixture as the flame has moved further upstream with the greater addition of H_2_. As a disturbance is superimposed on the fuel inlet, the temperature trace appears to follow a monotonic increase with the increase of H_2_ amplitude. However, this pattern is not observed as the CH_4_ amplitude was increased. Case 1y and case 3y show greater temperature readings and eventually result in flashback albeit case 2y which maintains stable operation with a lower temperature.

**Figure 13:**
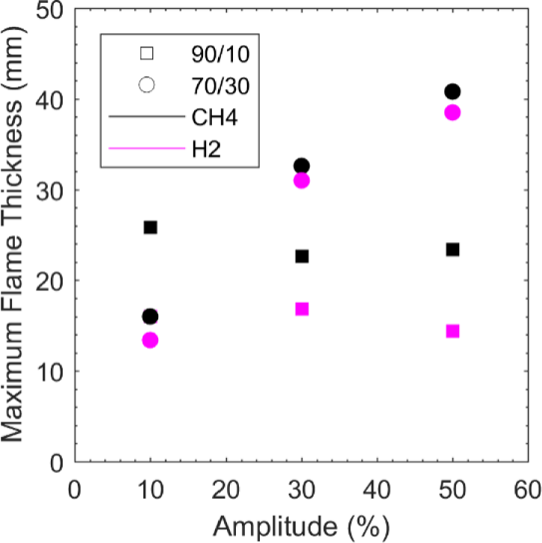
Maximum flame thickness vs base value amplitude.

Figure 13 depicts a correlation between the flame thickness and the variation of amplitude of hydrogen and methane in both CH_4_(90%)–H_2_(10%) and CH_4_(70%)–H_2_(30%) mixtures. Once again, the flame thickness in this figure was inferred through the image processing technique detailed in Section 2.3. In the CH_4_(90%)–H_2_(10%) mixture, it can be seen that as the methane amplitude is increased, mid-way, the flame thickness contracts 13% before posting a gradual increase of 4%. An inverse behavior is observed for hydrogen modulation, as the amplitude increases so do the flame thickness mid-way but then contracts at the maximum amplitude value. However, for the CH_4_(70%)–H_2_(30%) mixture a monotonic increase in flame thickness is detected for an increase in amplitude for both methane and hydrogen, approximately, 300% overall. The exact reason for this behavior is not immediately obvious, and it calls for further experimental and numerical studies.

## 4 Conclusions

A custom-designed porous burner was used in an experimental investigation of ultralean combustion to investigate the burner response to the oscillations superimposed on the fuel flow. The work was motivated by the possibility of temporal changes in the chemical composition of hydrogen containing fuels including blends of methane and hydrogen. Such variations can strongly influence flame stabilization and lead to flame extinction/flashback. It is therefore important to analyze the burner response to imposed oscillations on the fuel streams. CH_4_(90%)–H_2_(10%) and CH_4_(70%)–H_2_(30%) mixtures were incorporated as the fuel. The inlet sinusoidal disturbances were imposed on the hydrogen and methane flows by programmable MFCs. The thermal response of the system was monitored by using thermocouples positioned at the center of the burner in different axial locations and by detecting the flame motion using image processing techniques. It was found that under steady-state conditions, CH_4_(90%)–H_2_(10%) could operate as low as ϕ = 0.25. Additionally, the CH_4_(90%)–H_2_(10%) mixture produced greater carbon monoxide emissions when compared to the CH_4_(70%)–H_2_(30%) mixture. As both mixtures were subject to unsteady inlet flow of hydrogen and methane, the burner remained stable for the CH_4_(90%)–H_2_(10%) mixture when hydrogen was oscillated between 0 and 50% and for methane between 0 and 10% over a period of 60 s. Similarly, the burner remained stable for the CH_4_(70%)–H_2_(30%) mixture when hydrogen flow was fluctuated at 0–30% of its steady flow rate and methane fluctuated at 30% for the identical oscillatory period. For both mixtures by in large, it was observed that the flame movement corresponds to the dynamics of the induced disturbances at the inlet. The flame thickness within CH_4_(70%)–H_2_(30%) mixture was proven to be far greater when compared to the CH_4_(90%)–H_2_(10%) mixture as the hydrogen and methane flows were amplified. Lastly, both fuel mixtures were noted to be rather insensitive to hydrogen fluctuation beneath 30% amplitude.
